# 肺癌骨转移诊疗专家共识（2014版）

**DOI:** 10.3779/j.issn.1009-3419.2014.02.01

**Published:** 2014-02-20

**Authors:** 燕 孙, 忠震 管, 美琳 廖, 欣 于, 长利 王, 洁 王, 晓辉 牛, 远凯 石, 修益 支, 云鹏 刘, 孟忠 刘, 沂平 张, 跃 杨, 靖南 沈, 公琰 陈, 清华 周, 彩存 周, 其森 郭, 丽丽 唐, 建春 段, 军 梁, 英剑 章, 颖 程

**Affiliations:** 1 100021 北京，中国医学科学院肿瘤医院 Cancer Hospital, Chinese Aacademy of Medical Sciences and Peking Union Medical College, Beijing 100021, China; 2 510060 广州，中山大学肿瘤防治中心 SunYat-Sen University Cancer Center, Guangzhou 510060, China; 3 200030 上海，上海市胸科医院 Shanghai Chest Hospital, Shanghai, 200030, China; 4 100191 北京，北京大学第六医院精神科 Department of psychiatry, Peking University Sixth Hospital, Beijing 100191, China; 5 300060 天津，天津市肿瘤医院肺部肿瘤科 Department of Lung Cancer Surgery, Tianjin Cancer Hospital, Tianjin 300060, China; 6 100142 北京，北京大学肿瘤医院胸部肿瘤内科 Department of Thoracic Medical Oncology, Beijing Cancer Hospital, Peking University, Beijing 100142, China; 7 100035 北京，北京积水潭医院骨肿瘤科 Department of Orthopedic Oncology Surgery, Beijing Jishuitan Hospital, Beijing 100035, China; 8 100021 北京，中国医学科学院肿瘤医院肿瘤内科 Department of Medical Oncology, Cancer Hospital, Chinese Aacademy of Medical Sciences and Peking Union Medical College, Beijing 100021, China; 9 100053 北京，首都医科大学肺癌诊疗中心，首都医科大学宣武医院胸外科 Department of Thoracic Surgery, Beijing Lung Cancer Center, Beijing Xuanwu Hospital, Capital Medical University, Beijing 100053, China; 10 110001 沈阳，中国医科大学附属第一医院肿瘤内科 Departmentof Medical Oncology, the First Hospital of China Medical University, Shenyang 110001, China; 11 510060 广州，中山大学肿瘤医院放疗科 Department of Radiotherapy, Sun Yat-Sen University Cancer Center, Guangzhou 510060, China; 12 310022 杭州，浙江省肿瘤医院化疗中心 Department of Chemotherapy, Zhejiang Cancer Hospital, Hangzhou 310022, China; 13 100142 北京，北京大学肿瘤医院胸外科 Department of Thoracic Surgery, Beijing Cancer Hospital, Peking University, Beijing 100142, China; 14 510080 广州，中山大学附属第一医院骨肿瘤外科 Department of Orthopedic Oncology Surgery, the First Affiliated Hospital Sun Yat-Sen University, Guangzhou 510080, China; 15 150081 哈尔滨，哈尔滨医科大学附属肿瘤医院肿瘤内科 Department of Medical Oncology, Haerbin Medical University Cancer Hospital, Haerbin 150081, China; 16 300052 天津，天津医科大学总医院肺外科 Department of Thoracic Surgery, Tianjin Medical University General Hospital, Tianjin 300052, China; 17 200433 上海，上海市肺科医院肿瘤科 Departmentof Medical Oncology, Shanghai Pulmonary Hospital, Shanghai 200433, China; 18 250117 济南，山东肿瘤医院肿瘤内科 Department of Medical Oncology, Shandong Province cancer Hospital, Jinan 250117, China; 19 100142 北京，北京大学肿瘤医院康复科 Department of rehabilitation, Beijing Cancer Hospital, Peking University, Beijing 100142, China; 20 266003 青岛，青岛大学医学院附属医院肿瘤科 Department of Medical Oncology, the Affiated Hospital of Qingdao University, Qingdao 266003, China; 21 200032 上海，复旦大学附属肿瘤医院核医学科 Department of Nuclear Medicine, Fudan University Shanghai Cancer Center, Shanghai 200032, China; 22 130012 长春，吉林省肿瘤医院肿瘤内科 Department of Medical Oncology, Jilin Cancer Hospital, Changchun 130012, China

## 1 概述

原发性肺癌是我国最常见的恶性肿瘤之一^[1-4]^。2012年中国肿瘤登记年报显示：肺癌发病率和死亡率居全国众癌之首，且其发病隐匿，确诊时约50%为晚期（Ⅳ期），骨转移是主要的血行转移部位之一^[5]^。随着治疗方法和技术的进步，晚期肺癌患者的中位生存时间逐渐延长至1年左右^[6]^。患者生存获益的同时，发生骨转移及骨相关事件（Skeletal Related Events, SRE）的风险亦随之增高^[7-10]^。

骨转移常预示患者生活质量的下降和生存期的缩短。引起的SRE，如骨痛、病理性骨折、脊髓压迫、高钙血症及相关治疗带来的痛苦等，严重影响患者的生活质量。在控制原发疾病的同时，积极预防和治疗骨转移骨相关事件尤为重要。

在原发病的系统治疗基础之上，针对骨转移采取多学科综合治疗（mulitiple department treatment, MDT）模式，有计划、合理地制定个体化综合治疗方案，减少或延缓骨转移并发症及骨相关事件的发生，将有助于提高患者的生活质量。与以往恶性肿瘤骨转移诊疗共识不同，本共识特别增加了肺癌骨转移的心理治疗，强调肿瘤患者的身-心并重的整体治疗，符合生物-心理-社会医学模式，使患者最大程度获益。

## 2 发病率

肺癌骨转移发生率为30%-40%，研究显示甚至有50%的肺癌患者死后尸解发现有骨转移。肺癌骨转移后患者的中位生存时间仅6个-10个月^[11]^，经过治疗后1年生存率也仅为40%-50%。肺癌骨转移的好发部位在脊柱和躯干骨近端。发生于脊柱者占50%，股骨占25%，肋骨和胸骨占12%^[7, 12-14]^。

46%的肺癌骨转移患者并发骨相关事件SRE^[15]^。肺癌骨转移患者一旦发生SRE，将显著缩短患者生存期，有研究显示生存时间可缩短一半^[11]^。若合并严重骨相关事件，如高钙血症、病理性骨折、脊髓压迫等并发症，患者的生存将进一步缩短^[11, 12]^。

## 3 病理与发病机制

恶性肿瘤骨转移按病变特征可分为以下三种类型：溶骨型、成骨型和混合型^[12, 16]^。成骨型骨转移常见于前列腺癌、膀胱癌，约占骨转移的10%。溶骨型骨转移占70%，常见于肺癌和乳腺癌^[12, 13, 16]^。骨转移致SRE是影响骨转移患者生活质量和生存的直接因素。SRE发生危险性与恶性肿瘤类型相关。溶骨型病变为主的骨转移患者发生SRE危险性高。

肺癌骨转移主要是破骨细胞导致的骨吸收，大多表现为溶骨型病变^[17]^。肺癌细胞转移到骨后释放出可溶性介质，激活破骨细胞和成骨细胞。破骨细胞释放的细胞因子又进一步促进肿瘤细胞分泌骨溶解的介质，从而形成了恶性循环。应用抑制破骨细胞活性的药物，如双膦酸盐等可显著降低恶性骨转移瘤病灶内的破骨活动，降低由此引起的高钙血症和高尿钙症^[18]^。

## 4 临床表现

仅50%肺癌骨转移患者出现临床症状^[12]^。肺癌骨转移常伴有严重骨痛及骨相关事件（病理性骨折、脊髓压迫、高钙血症等）^[19, 20]^，不仅明显影响患者睡眠、情绪、日常生活能力，而且威胁患者的生存。

骨痛为骨转移最主要的临床症状。随着肿瘤增大至骨髓腔内压力>6.67 kPa出现骨痛，且随病情进展逐渐加重。肿瘤分泌的前列腺素、IL-2、TNF等疼痛介质及肿瘤侵犯骨膜、神经、软组织均可导致剧烈疼痛（疼痛程度评估见附件1）。

病理性骨折常为肺癌骨转移癌的首发症状。约1/3患者以骨转移癌为首发症状而无原发癌表现^[21]^。在此前，患者可全无自觉症状，甚至带瘤生存数月至数年。高钙血症是肺癌骨转移的致死原因之一。肺癌骨转移晚期还可出现乏力、消瘦、贫血、低热。

肺癌患者相关心理痛苦主要表现为焦虑、抑郁、失望及孤独等。因此，患者心理需求是大量的，如安全感、爱与被爱、理解、自尊等。如果这些需求得不到确认和较好的满足，就不可能获得疼痛及其他症状全身心的缓解。

## 5 诊断

### 5.1 高危因素

原发肺癌病史的患者出现以下列任何情况均可视为骨转移的高危人群，需进行骨转移相关检查：①骨痛/骨折；②脊髓或神经受压症状；③碱性磷酸酶升高；④高钙血症^[22]^。

### 5.2 诊断方法

对怀疑有骨转移的肺癌病人推荐病人进行以下检查：

对怀疑有骨转移的肺癌病人推荐病人进行以下检查：

1放射性核素骨扫描（ECT）检查或PET-CT检查

2 ECT检查阳性的部位行X线平片

3 ECT检查阳性的部位行CT及/或MRI检查

4血常规、肌酐、肝功能、电解质、血清钙等生化指标检查

#### 5.2.1 放射性核素骨扫描（ECT）

ECT是骨转移的首选筛查方法^[23, 24]^，能够早期发现发生在骨骼中的成骨、溶骨或混合破坏性骨破坏的转移性病灶。具有灵敏度高、全身一次成像不易漏诊的优点，但由于不仅骨转移瘤可以在放射性核素骨扫描检查中表现出阳性结果，其他的骨病变也可以在放射性核素骨扫描检查中表现出阳性结果，因此该检查存在特异度较低的缺点^[25]^。PET- CT不仅可以提供全身骨骼受累情况，还可以断层显像显示骨破坏的情况，其缺点是价格昂贵^[26]^。

#### 5.2.2 X线

X线是最常规的骨骼检查方法，可以显示骨骼局部的全貌，是骨科必须的检查方法。早期病变普通X线检查难以发现，敏感性低（仅44%-50%左右）^[7, 27]^，常比ECT显示骨转移灶晚3个-6个月，骨破坏未累积皮质时，易被高密度皮质掩盖而漏诊。但其特异性高、操作简单、能基本显示骨质密度变化且费用低廉，可对其他影像检查发现的骨质异常进行进一步确认。故仍是诊断骨转移的主要诊断工具^[22]^。

#### 5.2.3 CT/增强CT

转移瘤的主要特点是破坏掉骨骼的正常结构，然后由肿瘤组织替代、占据被破坏的骨结构。CT可以显示骨骼的细微结构，是目前最高空间分辨率的影像，为ECT阳性患者的确诊性检查，以明确是否有骨破坏、并了解破坏程度；增强CT可以显示占据被破坏骨结构中的组织是否具有较正常组织更丰富的血供，而这正是肿瘤组织的特征。较X线、常规CT可获得更多骨微细变化及周围软组织病变信息。

#### 5.2.4 MRI检查

诊断骨转移的敏感性和特异性均高，能通过横断、冠状、矢状位多角度观察，能详细了解解剖结构而广泛应用于临床。恶性肿瘤通过血液循环转移至骨，首先先侵犯骨髓（高信号），与骨皮质破坏区形成良好对比，对早期病理改变有较高敏感性，但应与化疗后骨髓局灶性反应性改变相鉴别。应当注意的是许多髓腔MRI信号改变并不是由于肿瘤侵及造成。MRI显示骨髓和软组织解剖清晰，伴脊柱神经压迫症状时首选MRI。但其价格较贵、扫描范围局限，需恰当选择。当判断骨转移时ECT结合X线仍不能确定时，可行MRI检查提供间接证据。

#### 5.2.5 骨活组织检查

病理学是诊断肺癌骨转移的金标准。其原则和指征：如肺癌诊断明确，且全身多发骨破坏（椎体、骨盆、长骨），活检为非必须操作；肺癌诊断明确，但仅出现孤立的骨破坏灶，应积极穿刺活检，明确诊断。骨转移病灶的活检遵循肌肉骨骼系统肿瘤的活检原则，穿刺针抽取肿瘤组织，偶有切开活检，活检切口需与将来手术切口一致，有利于切除活检的污染伤口或穿刺针道。骨骼在取活检开窗时，尽可能取圆形窗，以减少病理骨折发生的危险。活检后填充骨水泥，减少出血。术后压迫止血，忌放置引流管，以免造成肿瘤局部播散。为证明取材部位正确性，肢体活检应在影像增强仪下进行；躯干、脊柱椎体、腰骶部病变应在CT引导进行。骨活检过程中需注意避免造成病理性骨折。

#### 5.2.6 骨代谢的生物化学标记（Bone Biomarkers）

可反映骨转移过程中骨吸收和形成的速度，提示骨破坏和修复程度。是近期发现的具有潜在的用于诊断及监控疾病进展的新技术，但因目前尚无前瞻性研究，除碱性磷酸酶（ALP）外，暂不建议临床常规使用。①反映溶骨代谢水平的标记：Ⅰ型胶原羧基末端肽（ICTP）、Ⅰ型胶原N末端肽（NTX）、Ⅰ型胶原α1羧基末端肽（CTX）、骨唾液蛋白（BSP）等；②反映成骨代谢水平的标记：骨特异性碱性磷酸酶（BALP）、碱性磷酸酶（ALP）、Ⅰ型溶胶原N末端肽（PINP）等^[28, 29]^。

### 5.3 诊断标准

肺癌骨转移的诊断应满足以下两个条件之一：

（1）临床或病理诊断肺癌，骨病变活检符合肺癌转移；

（2）肺癌病理诊断明确，具有典型的骨转移影像学表现。

### 5.4 诊断流程

**Figure:**
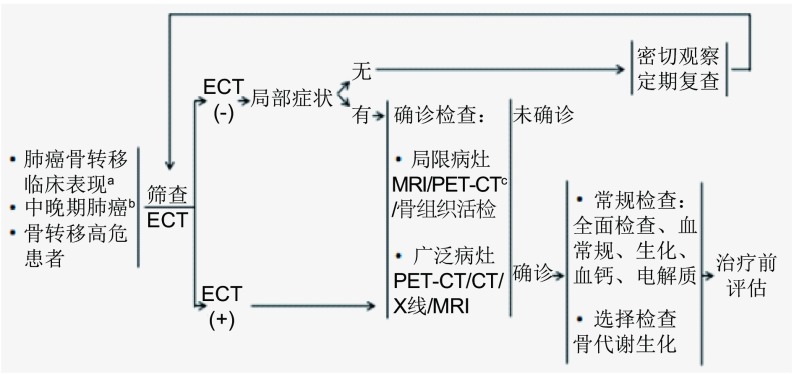
a.骨转移的临床表现:骨疼痛、活动障碍、病理性骨折、脊髓压迫及脊神经压迫、高钙血症等; b.中晚期恶性肿瘤及高风险发生骨转移的恶性肿瘤; c.由于该检查费用昂贵, 因此不推荐作为常规检查。

## 6 治疗

治疗肺癌骨转移的目标是提高生活质量、延长生命、缓解症状及心理痛苦、预防或处理病理性骨折、解除神经压迫等骨相关事件。肺癌出现骨转移时即为全身性疾病，应采取以全身治疗为主的综合治疗方式，包括：肺癌（原发病）的系统治疗（化疗及分子靶向治疗）、放疗、手术、止痛、双膦酸盐和心理支持治疗。

治疗原则：以全身治疗为主，其中化疗、分子靶向治疗可作为肺癌的抗肿瘤治疗方式，具体原则可参照原发性肺癌诊疗规范（2011版）。合理的局部治疗可以更好的控制骨转移相关症状，其中手术是治疗孤立骨转移灶的积极手段，而放射治疗也是有效的局部治疗手段。双膦酸盐可以预防和延缓SRE的发生。对症止痛治疗可明显改善患者的生活质量。应根据患者的机体状况、肿瘤病理学类型、病变累及范围（临床分期）和发展趋势，采取多学科综合治疗（MDT）模式，有计划、合理的制定个体化综合治疗方案。

主要抗癌方法的适应症：

化疗及分子靶向治疗：多发骨转移灶

放疗：孤立骨转移灶（体外放射治疗）、多发骨转移灶（放射性核素治疗）、脊髓外压迫、外周神经肿瘤性压迫或侵犯所致疼痛或功能障碍

手术：病理性骨折及脊髓压迫固定术

镇痛药：骨痛

双膦酸盐：诊断明确的骨转移

心理治疗：出现抑郁或焦虑的患者

### 6.1 化疗及分子靶向治疗

#### 6.1.1 化疗

肺癌骨转移患者治疗前一般情况评估见附件2（PS评分）。

化疗适应症：PS评分≤2分（附件3，ZPS评分，5分法），重要器官功能可耐受化疗。对于小细胞肺癌（small cell lung cancer, SCLC）的化疗PS评分可放宽至3分。

含铂双药联合化疗是晚期肺癌患者的标准一线方案，在缓解率和生存期方面均显著优于单药方案。研究表明，对肺癌原发灶有效的化疗药物（联合铂类的第三代双药方案，如长春瑞滨联合铂类-NP方案、吉西他滨联合铂类-GP方案、紫杉醇联合铂类-TP方案、多西他赛联合铂类-DP方案），对骨转移灶并非一定有效，故骨转移灶的局部治疗仍是不可或缺的治疗方式；三药方案能够提高化疗的有效率，但毒性有所增加，且不能改善患者总生存；非铂方案可作为不能耐受铂类毒性患者的一线方案。全身化疗可改善患者一般情况，提高生活质量。对于骨转移，一般需同时联合双膦酸盐药物，具体参考见《NCCN非小细胞肺癌临床实践指南》中复发和转移一章及《原发性肺癌诊疗规范（2011版）》^[22, 30]^，鼓励患者参加临床试验。

#### 6.1.2 分子靶向治疗

肺癌的分子靶向治疗是针对可能导致细胞癌变的驱动基因，从分子水平上阻断肿瘤信号传导通路，从而抑制肿瘤细胞生长，诱导凋亡，甚至使其完全消退的全新的生物治疗模式。根据药物的作用靶点，肺癌常用的分子靶向治疗药物包括：

(一)以EGFR突变为靶点表皮生长因子受体酪氨酸激酶抑制剂（Epidermal Growth Factor Receptor Tyrosine Kinase, EGFR-TKI），如厄洛替尼、吉非替尼、埃克替尼，在具有EGFR基因敏感突变的非小细胞肺癌患者中的疗效已经得到肯定。对携带EGFR基因敏感突变的非小细胞肺癌骨转移患者，EGFR-TKI可作为一线治疗方案。

(二)以EML4-ALK融合基因为靶点中国非选择性非小细胞肺癌人群EML4-ALK（棘皮动物微管相关蛋白样4-间变性淋巴瘤激酶，Echinoderm Microtubule -Associated-Protein-Like 4-Anaplastic Lymphoma Kinase）融合基因发生率约为4%。克唑替尼是ALK（间变性淋巴瘤激酶，anaplastic lymphoma kinase）、MET和ROS-1（C-ROS原癌基因1酪氨酸激酶，C-Ros Oncogene 1 receptor tyrosine kinase）酪氨酸激酶的抑制剂，对有EML4-ALK融合基因的晚期非小细胞肺癌患者的疾病控制率可达60%-70%，已经成为继EGFR-TKI后又一种具有明确分子靶点和疗效预测标志的靶向药物。

(三)以VEGF为靶点的治疗贝伐单抗是人源化抗血管内皮生长因子受体（Vascular Endothelial Growth Factor Receptor, VEGFR）的单克隆抗体，可以与VEGF结合，使之不能与受体结合，抑制肿瘤新生血管形成。在非鳞型非小细胞肺癌贝伐单抗与化疗联合应用能够提高非鳞型非小细胞肺癌的疗效并延长患者生存。贝伐单抗联合含铂双药化疗药物是目前晚期非鳞型非小细胞肺癌的标准一线治疗方案之一。

### 6.2 放射治疗

放射治疗是肺癌骨转移有效的治疗方法之一，能够减轻/消除症状、改善生活质量、延长生存，还能预防病理性骨折和脊髓压迫的发生及缓解脊髓压迫症状。放射治疗包括外照射和放射性核素治疗两类。

#### 6.2.1 体外放射治疗

体外放射治疗是肺癌骨转移姑息性放疗的首选方法，对经化疗和双膦酸盐治疗后仍无法缓解的顽固性疼痛、椎体不稳、即将发生病理性骨折和脊髓压迫症的患者（对于已有明显脊髓压迫可先请神经外科确定有无手术指征），局部放疗可迅速有效地缓解骨破坏和软组织病变导致的疼痛。对于长骨骨折病人，放疗可有效控制疼痛，并有可能促进骨折愈合。双膦酸盐阻止肿瘤细胞由G2期和M期向S期转换，使细胞停滞于放疗敏感的细胞周期时段延长，故可常规联用双膦酸盐以增强骨转移灶对放疗的敏感性^[31, 32]^。

(一)治疗前评估患者全身症状及其他部位肿瘤情况评价：①患者全身状况及其他部位肿瘤情况评价：1）PS评分 < 2，全身病变稳定：较高剂量较长时间；2）PS评分2-3，其他部位进展：较低剂量较短时间；②骨转移部位：1）承重骨即使无症状亦可预防照射；2）非承重骨其他治疗手段缓解疼痛症状无效、影响功能。

(二)体外放射治疗适应症：①有疼痛症状的骨转移灶，缓解疼痛及恢复功能；②选择性的用于负重部位骨转移的姑息性放疗（如脊柱或股骨转移）^[12, 33]^。

骨痛需要放疗的SRE定义：①非承重骨的骨转移，伴骨痛（VAS≥4分），经中度止痛药无效而接受放疗属于SRE；②承重骨骨转移，伴疼痛（VAS≥4分）接受放疗属于SRE；③承重骨骨转移无疼痛，但有明显骨质破坏而接受放疗则属于伴随治疗。

(三)体外放射治疗常用剂量及分割方法（选择下列方法之一）：①300 cGy/次，共10次；②400 cGy/次，共6次；③400 cGy/次，共5次；④800 cGy/次，单次照射（顽固性疼痛、已发生或即将发生的病理性骨折的患者，推荐剂量为8 Gy/次-10 Gy/次）。

(四)单次放射及多次放射的适应症：单次放射适用于：①用于非中线骨转移；②行动不便急需解决骨痛的患者。多次放射适用于：①因其他治疗止痛无效因疼痛需要放疗的骨转移患者；②有骨折风险患者。对大多数无椎体骨或重要结构骨转移的初治骨转移瘤患者，可推荐单次大剂量放疗^[33]^。但再放疗和病理性骨折的发生率高于分次放疗。推荐单次放疗用于活动及搬运困难的晚期肺癌骨转移患者^[34]^。

(五)疗效评价：①疼痛改善程度：完全缓解，指疼痛明显减轻或基本消失，恢复正常活动，基本可以不用止痛药；部分缓解，指疼痛减轻，止痛药使用明显减少。因骨转移所导致的功能障碍部分缓解；无效，疼痛略减轻或无明显缓解，止痛药物剂量不能减少。②影像学检查。

#### 6.2.2 放射性核素治疗

放射性核素治疗是肺癌骨转移的一种有效的治疗手段。放射性核素治疗应严格掌握适应症，不能优先选择。主要是由于部分患者放射性核素治疗后会出现明显的骨髓抑制且恢复较慢，影响化疗等后续全身治疗。因此，放射性核素治疗前应影像学确认，多学科共同评估，为患者选择合适的治疗及恰当的治疗时机。

(一)分类目前骨转移癌放射性核素治疗的常用药物包括：89Sr和153Sm。89Sr：是骨转移内科放射治疗中最常用的核素药物，半衰期50.6天，组织中最大射程6.67 mm，发射纯β射线，化学性质类似于钙，聚集在成骨活跃的部位。153Sm：半衰期46.3小时，组织中射程3.4 mm，发射β及γ射线。

(二)适应症及禁忌症适应症：①骨转移肿瘤患者伴有明显骨痛；②经临床、CT或MRI、全身骨显像和病理确诊多发骨转移肿瘤，尤其是前列腺癌、乳癌和肺癌骨转移患者且全身骨ECT显像病灶处有放射性浓聚；③原发性骨肿瘤未能手术切除或残留者，或伴转移者；④WBC>3.5×10^9^/L，PLT>80×10^9^/L。禁忌症：①骨显像示转移灶仅为溶骨型冷区；②严重骨髓、肝、肾功能障碍的患者；③近期（6周内）进行过细胞毒药物治疗的患者。

(三)注意事项：①与最后一次化疗或放疗时间间隔一个月为宜；②因骨髓抑制风险较高，恢复较慢（约12周），治疗前行血常规及肝肾功能等常规检查；③治疗前需取得CT或MRI、全身骨显像或行局部活检组织学证实骨转移。

(四)常用剂量及方法89Sr的常用剂量为1.48 MBq（40 μCi）/kg体重，或（111-148）MBq（3 mCi-4 mCi）/次，3个-6个月后可重复应用；153Sm的常用剂量为（18.5-22）MBq/kg体重（总量不超过40 mCi），2个-3个月后可重复应用。给药方法：一次静脉注射。

(五)疗效评价对肺癌骨转移尤其是多发性转移灶有明显疗效，可使部分骨转移灶缩小或消失。通过破坏肿瘤组织缓解骨痛，但镇痛效果起始时间不定，多在用药后1-2周开始起效。

### 6.3 手术治疗

肺癌易于发生骨转移，由于治疗的进步，其总体中位生存期较前不断提高，同时出骨的合并症发生率亦增高，如不对骨转移灶进行治疗，病人生存质量将受到极大影响。无论肿瘤细胞直接破坏骨质，还是由于肿瘤骨转移所致破骨细胞活性增加而骨质下降，都会出现肿瘤包块形成、骨强度下降^[35, 36]^。溶骨破坏的结果就是运动系统功能受损。“生命在于运动”，肺癌骨转移的治疗与原发病变的治疗一样重要。

#### 6.3.1 外科治疗的主要目标

1）获得骨转移病灶的组织学诊断，便于肿瘤的进一步内科治疗。

2）缓解疼痛。

3）防止或固定骨折。

4）恢复或维持肢体的运动。

5）便于综合治疗。

6）便于护理。

7）脊柱病变，明确诊断，治疗原发肿瘤。

8）提高生存质量。

9）减少或避免运动系统功能受损所引发的并发症，间接延长生存期。

#### 6.3.2 外科治疗原则

1) 预计病人可存活三个月以上。

2) 全身状况好，能够耐受手术创伤及麻醉。

3) 预计外科治疗后较术前更好的生活质量，能够立即活动，要有助于进一步治疗和护理。

4) 预计原发肿瘤治疗后有较长的无瘤期。

5) 经全身治疗后，溶骨病灶趋于局限、骨密度增高。

6) 孤立的骨转移病灶。

7) 病理骨折风险高者。

#### 6.3.3 外科干预治疗时机

1) 有恶性肿瘤病史，影像学及组织学检查为单发骨转移者。

2) 负重骨出现平片可见的骨破坏。

3) 保守治疗后，骨破坏仍继续加重的患者。

4) 保守治疗后，疼痛仍继续加重的患者。

5) 保守治疗后，运动系统功能仍不能恢复者。

6) 已经出现病理骨折的患者。

7) 有神经压迫症状者。

8) 脊柱溶骨性破坏，出现截瘫危险性大的患者。

9) 放、化疗治疗不敏感骨转移灶，如肾癌骨转移等。

#### 6.3.4 手术适应症

(一)负重长管状骨内固定的适应证：

1) 即将发生骨折。

2) 已发生骨折。

3) 病变直径>2.5 cm。

4) 病变>50%皮质。

5) 完全溶骨。

6) 负重下疼痛。

7) 放疗后疼痛。

在病理骨折前进行外科治疗，最能极大提高生存质量，使病人免受骨折之苦。预防性内固定的治疗比较骨折的治疗要简单、安全的多。应用病理骨折风险预测系统可以指导预防性内固定的实施，评分7分以下可暂时不考虑手术，而评分7分以上有高骨折风险，应进行手术治疗。

(二)脊柱转移癌：

1) 神经功能受损。

2) 脊柱不稳定。

3) 即将发生骨折。

4) 疼痛。

(三)骨盆转移癌：

1) 髋臼即将或已发生病理骨折。

2) 顽固性疼痛。

3) 对侧即将发生骨折而需外科治疗。

#### 6.3.5 外科禁忌症

对于下列因素应考虑非手术治疗：

1) 高度恶性侵袭性原发肿瘤。

2) 预计原发肿瘤治疗后无瘤生存期短于3个月。

3) 经全身治疗后，骨转移灶的溶骨破坏未见好转。

4) 全身多发骨破坏。

5) 涉及多器官转移。

6) 全身一般条件差，有手术禁忌症。

#### 6.3.6 外科治疗的基本思想及关键点

(一)骨转移瘤的治疗需多学科协作，骨科医师、肿瘤内科医师及放疗科医师应分工明确。在制定治疗方案时应考虑的因素包括：预期寿命、肿瘤的类型和分期、有无内脏转移、Karnofsky和Burchenal患者状况评分、发现原发灶至出现转移灶的时间、病理骨折的风险以及对化疗、激素疗法和放疗敏感程度的预测。其外科治疗的基本思想：无需期待骨愈合，固定要即刻坚定。

(二)长管状骨转移癌外科治疗关键点：

1) 内植物坚强、稳定。

2) 包括所有骨强度降低区。

3) 尽可能切除肿瘤。

4) 内植物寿命长于病人寿命。

(三)脊柱转移癌外科治疗关键点：

1) 病变多发生于椎体，应采用前入路。

2) 对病变部位尽量切除肿瘤，彻底解除对脊髓的压迫。

3) 避免单纯后路椎板减压术，这会加重脊柱的不稳定性。

4) 前路重建纠正后突畸形，后路重建维护脊柱稳定性。

5) 椎体成形术并不完全适于椎体转移癌的治疗，风险大，效果不确定。

(四)骨盆转移癌外科治疗关键点：

1) 未累及髋臼的髂骨病变，应用内固定及骨水泥加强应力传导区。

2) 累及髋臼的髂骨病变，应行全髋关节置换，并应用内固定及骨水泥加强应力传导区。

3) 非应力传导区病变（耻、坐骨），可行单纯切除。

随着肺癌骨转移多学科综合治疗模式的发展，我们需要提高肿瘤内科医师对骨转移癌可以并需要外科治疗的认识，提高对骨转移癌施行外科治疗的骨科医生的肿瘤学知识，建立较为完善的骨转移癌外科治疗综合评估系统，在多学科综合治疗指导下选择恰当的病人进行恰当的治疗。肿瘤科内科的全身治疗，骨科医师的骨转移癌外科治疗，放疗科医师的骨转移癌术后放疗支持，都将有助于提高病人的生存质量及总生存期。

### 6.4 镇痛治疗

在当前肿瘤的综合治疗时代，由于癌痛的复杂性，对癌痛的处理应采用综合治疗手段。即根据癌痛患者的机体状况、疼痛的不同程度、性质及原因，合理、有计划地应用现有的治疗手段，尽可能缓解癌痛及并发症、改善生活质量、提高患者接受抗癌治疗的依从性、进一步延长生存期，提高生存率。

癌痛治疗原则：①综合治疗；②从无创性和低危险性方法开始，然后再考虑有创性和高危险性方法。

癌痛综合治疗

药物治疗是缓解肺癌骨转移疼痛的主要方法之一。镇痛治疗应遵循世界卫生组织（WHO）癌症三阶梯止痛治疗指导原则见附件4^[40, 41]^。镇痛药物可与双膦酸盐药物或放疗、手术等方法联合，以最大限度缓解肺癌骨转移的疼痛。

癌痛综合治疗

1.癌痛综合评估（见附件1）

2.姑息性抗癌治疗及全身性非阿片类/阿片类镇痛药物

3.全身阿片类药物治疗弊大于利时，考虑非侵袭性干预措施^[37-39]^

恰当姑息性抗癌治疗

加用非阿片类药物

加用辅助药物

应用认知和行为干预措施

借助矫形疗法、其他物理疗法和社会心理干预

4.全身阿片类药物治疗弊大于利时，考虑侵袭性干预措施

区域性止痛技术

神经阻滞术

神经切断术

5.若上述方法无效时，应用镇静剂等辅助药物协助处理顽固性疼痛

### 6.5 双膦酸盐治疗

双膦酸盐是肺癌骨转移的基础用药，可以和常规抗肿瘤治疗（化疗、靶向治疗、放疗、放射性核素治疗和手术治疗）联合使用。

#### 6.5.1 药理作用及机制

双膦酸盐是焦膦酸盐分子的稳定类似物，以P-C-P基团取代焦磷酸盐结构中的P-O-P基团，改变焦磷酸盐的理化性质，增加其对水解酶的稳定性，改变其生物学性质及毒理作用。其中一条侧链使钙离子晶体与骨矿化基质（羟磷灰石）高度亲和，另一条侧链的差别使不同的双膦酸盐抗骨吸收的能力不同。新一代双膦酸盐（如伊班膦酸）较第一代双膦酸盐羟乙膦酸的体外作用强1, 000至100, 000倍^[17]^。研究表明双膦酸盐治疗骨转移的机制包括：1）可以被破骨细胞选择性吸收，并选择性抑制破骨细胞活性，诱导破骨细胞凋亡，从而抑制骨吸收；2）抑制破骨细胞成熟；3）抑制成熟破骨细胞的功能；4）抑制破骨细胞在骨质吸收部位的聚集；5）抑制肿瘤细胞扩散、浸润和黏附于骨质。双膦酸盐能抑制破骨细胞对骨小梁的溶解和破坏，因此能阻止肿瘤转移引起的溶骨型病变、减少骨吸收、减轻骨痛及由骨转移所致的高钙血症及其他骨相关事件^[42, 43]^。另外，已有多项研究显示，部分双膦酸盐对癌细胞有直接抗肿瘤作用，抑制肿瘤细胞浸润和骨基质的粘附性，阻断肿瘤细胞释放破坏骨质释放的细胞因子和生长因子，并可诱导肿瘤细胞凋亡^[17, 44-47]^。

#### 6.5.2 临床应用

第一代双膦酸盐药物（羟乙膦酸、氯膦酸）和第二代双膦酸盐药物（帕米膦酸）能改善肿瘤骨转移患者疼痛、控制病情、预防骨转移的并发症和提高患者生活质量方面。第三代双膦酸盐药物伊班膦酸钠（邦罗力）、唑来磷酸在此基础上，还能显著降低恶性肿瘤骨转移的高钙血症，增加骨质密度，减少骨代谢紊乱。对于骨转移伴严重疼痛的患者，伊班膦酸负荷剂量可快速缓解肿瘤骨转移患者的疼痛^[48]^。双膦酸盐治疗骨转移的剂量和给药方案见7.6双膦酸盐治疗推荐^[12]^。

#### 6.5.3 适应症

肺癌患者明确诊断骨转移后，如无双膦酸盐应用禁忌症，均推荐应用双膦酸盐治疗。包括以下几种：①骨转移引起的高钙血症；②骨转移引起的骨痛；③ECT异常，X线或CT、MRI证实骨转移；④ECT异常，X线正常，但CT或MRI显示骨破坏；⑤无骨痛症状，但影像学诊断为骨破坏。下列情况不推荐使用双膦酸盐：①ECT异常，X线正常，CT或MRI也未显示骨破坏；②存在骨转移风险（LDH或ALP增高）的患者。

#### 6.5.4 用药时间及停药指征

一旦确诊肺癌骨转移应即刻应用双膦酸盐。研究证明双膦酸盐用于转移性肿瘤的中位时间为9个-18个月。因此，除非不能耐受该类药物的不良反应或出现禁忌症，应推荐至少应持续用药9个月以上，并根据患者获益情况考虑是否长期用药。停药指征：①用药过程中检测到与双膦酸盐治疗相关的严重不良反应；②治疗过程中出现肿瘤恶化，或出现其他脏器转移并危及患者生命；③继续用药不能获益。另外，研究表明患者治疗期间出现骨痛加重或SREs时，继续接受唑来磷酸治疗，可以减少再次发生SREs的风险，因此在应用某种双膦酸盐治疗过程中即使发生SREs仍建议继续用药，换药是否获益还有待更多的临床研究结果证实。

#### 6.5.5 不良反应及用药注意事项

双膦酸盐有较好的耐受性，主要不良反应包括：流感样症状（骨痛、发热、疲乏、寒战及关节痛和肌痛）、不需治疗的无症状血浆磷酸盐水平降低、低钙血症、肾功能损害、颌骨坏死（ONJ）等，偶有注射部位的轻度反应。很少有因不良反应而中断治疗，未见长期不良反应^[17]^。用药注意事项包括：①用药前监测患者血清电解质水平，重点关注血肌酐、血清钙、磷酸盐和镁等指标；②选择药物应考虑患者的一般状况、疾病的总体情况及同时服用的其他药物；③双膦酸盐可与化疗、靶向治疗、放疗等常规抗癌治疗及镇痛药联用；④用药期间应定期（3个-6个月）监测血钙，长期使用双膦酸盐应注意每日补充500 mg钙和适量维生素D；⑤用药期间应定期（3个-6个月）监测肾功能，肌酐清除率>30 mL/min的患者，除口服氯膦酸盐和伊班膦酸无需调整剂量外，其他双膦酸盐应根据产品说明书进行减量或延长输注时间^[48-51]^；⑥对少数患者长期使用双膦酸盐后有发生颌骨坏死的风险（由高到低为唑来膦酸、帕米膦酸、阿仑膦酸、利塞膦酸、伊班膦酸^[52, 53]^），应在用药前进行口腔检查，并进行适当的预防性治疗；用药期间应注意口腔卫生，尽量避免包括拔牙在内的口腔手术；如出现牙龈肿痛应停用，必要时下颌骨摄片评估风险。如治疗期间无诱因出现相关症状或体征，应尽早联系专科处理。增加下颌骨坏死风险的其他因素包括化疗、使用糖皮质激素以及口腔卫生差合并牙周疾病和牙周脓肿。⑦静脉应用时需注意急性期反应，发生率由高到低为唑来膦酸、帕米磷酸、伊班膦酸，可预防性或治疗性使用镇痛药缓解，无需停药^[53, 54]^。鉴于可能存在上述风险，建议临床医生在使用双膦酸盐药物时密切监护患者健康状况，应针对患者不同状况调整治疗方案，最大程度的保障患者的用药安全。

### 6.6 心理支持治疗

根据骨转移姑息治疗的基本原则，应针对骨转移及其相关并发症提供最佳支持治疗和症状治疗，需要肿瘤临床医生与心理精神科医生建立好一个多学科合作团队。近年来，癌症的治疗已经逐渐走向整合性疗法，也就是除了正统治疗以外，还搭配辅助与替代疗法（complementary and alternative medicine, CAM），强调身-心并重的整体治疗，癌症的治疗不仅要有好的治疗结果，还需要有好的治疗过程。

#### 6.6.1 与肺癌及骨转移相关的心理精神症状的处理

有研究提示在癌症患者中，心理痛苦的总患病率为35.1%，不同类型癌症间存在差异，其中肺癌患者的心理痛苦患病率最高，达到43.4%^[55]^。焦虑和抑郁是癌症患者的两个主要症状，有调查表明，肺癌患者亚临床抑郁35.3%，临床抑郁17.9%；亚临床焦虑48.3%，临床焦虑25.7%^[56]^。没有达到临床诊断的心理痛苦可由临床医护人员给予相应的心理支持和患者教育以降低患者对疾病进展的恐惧和紧张程度，适应疾病的状态。但是出现临床诊断意义的焦虑和抑郁一般需要心理及精神科医师的会诊和合作指导。

#### 6.6.2 心理治疗的主要内容及方法

心理社会干预可以有效缓解癌症患者的心理痛苦并改善总体生活质量。认知-行为治疗、支持性心理治疗以及家庭-夫妻治疗是最核心的3种心理治疗方式。癌症患者焦虑、抑郁的常见症状及治疗方法见附件5。

#### 6.6.3 精神药物干预

研究表明抗抑郁药与抗焦虑药物可用于癌症患者的焦虑与抑郁症状^[57-59]^。常用癌症患者的主要抗焦虑和抗抑郁药物用法及注意事项参见附件6和附件7。

### 6.7 肺癌骨转移治疗流程

## 7 诊疗推荐

### 7.1 诊断推荐

对怀疑有骨转移的肺癌病人推荐病人进行以下的检查：

1.放射性核素骨扫描（ECT）检查或PET-CT检查

2. ECT检查阳性的部位行X线平片

3. ECT检查阳性的部位行CT及/或MRI检查

4.血常规、肌酐、肝功能、电解质、血清钙等生化指标检查

**Figure:**
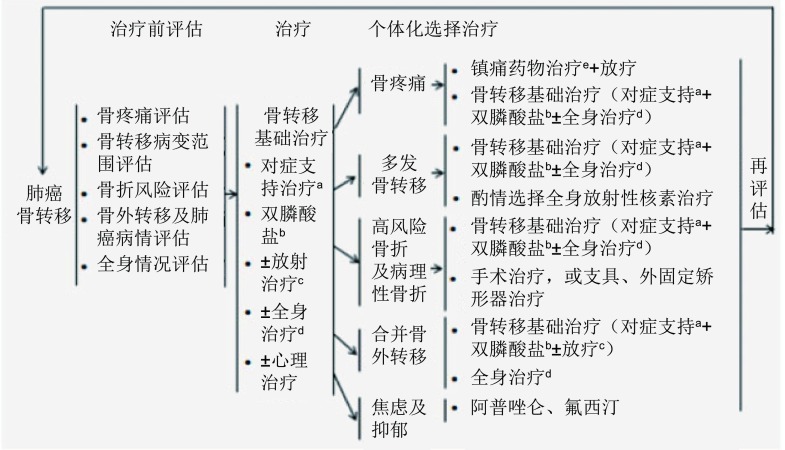
a、改善功能状态和生活质量的对症支持治疗；b、参见双膦酸盐治疗方案；c、参见放射治疗原则及方案；d、全身治疗包括：化疗、分子靶向治疗等，根据肿瘤病情及全身情况来决定抗肿瘤全身治疗；e、遵循WHO癌痛治疗原则。

### 7.2 化疗及分子靶向治疗推荐

含铂化疗方案或联合贝伐单抗是非小细胞肺癌骨转移的标准一线治疗方案。对携带EGFR敏感性突变或ALK融合基因的患者，推荐尽早使用EGFR-TKI或克唑替尼治疗。

### 7.3 放射治疗推荐

骨转移姑息性放射治疗及选择

1.体外照射：局部或区域放疗，骨转移放射治疗的常规放疗方法体外照射适应证：①有骨疼痛症状的骨转移灶，缓解疼痛及恢复功能；②选择性地用于负重部位骨转移的预防性放疗（如脊柱或股骨转移）。

体外放疗常用的剂量及分割方法：①每次300 cGy，共10次；②每次400 cGy，共6次；③每次400 cGy，共5次；④每次800 cGy，单次照射。

放疗可常规联合双膦酸盐治疗。

2.放射性核素：全身性内照射放疗，是肺癌骨转移的一种有效的治疗手段

目前骨转移癌放射性核素治疗的常用药物包括：89Sr和153Sm。

适应症：①经临床、CT或MRI、全身骨显像和病理确诊多发骨转移肿瘤，尤其是前列腺癌、乳癌和肺癌骨转移患者；且全身骨ECT显像病灶处有放射性浓聚；②骨转移肿瘤患者伴骨痛；③原发性骨肿瘤未能手术切除或残留者，或伴转移者；④ WBC>3.5×10^9^/L，PLT>80×10^9^/L。

禁忌症：①骨显像示转移灶仅为溶骨型冷区；②严重骨髓、肝、肾功能障碍的患者；③近期（6周内）进行过细胞毒素治疗的患者。

常用剂量及方法：89Sr的常用剂量为1.48MBq（40μCi）/Kg体重，或111-148MBq（3-4mCi）/次，3-6个月后可重复应用；153Sm的常用剂量为18.5-37MBq（40μCi）/Kg体重，必要时每月1次，连续给药。给药方法：一次静脉注射。

注意：该治疗发生骨髓抑制的风险较高，且恢复较慢（约12周）。

### 7.4 手术治疗推荐

1.负重长管状骨内固定的适应证：

① 即将发生骨折；②已发生骨折；③病变直径> 2.5 cm；④病变> 50%皮质；⑤完全溶骨；⑥负重下疼痛；⑦放疗后疼痛

2.脊柱转移癌：

① 神经功能受损；②脊柱不稳定；③即将发生骨折；④疼痛。

3.骨盆转移癌：

① 髋臼即将或已发生病理骨折；②顽固性疼痛；③对侧即将发生骨折而需外科治疗

4.外科治疗原则：

预计病人可存活三个月以上；全身状况好，能够耐受手术创伤及麻醉；预计外科治疗后较术前更好的生活质量，能够立即活动，要有助于进一步治疗和护理；预计原发肿瘤治疗后有较长的无瘤期；经全身治疗后，溶骨病灶趋于局限、骨密度增高；孤立的骨转移病灶；病理骨折风险高者。

5.外科禁忌症：

对于下列因素应考虑非手术治疗：高度恶性侵袭性原发肿瘤；预计原发肿瘤治疗后无瘤生存期短于3个月；经全身治疗后，骨转移灶的溶骨破坏未见好转；全身多发骨破坏；涉及多器官转移；全身一般条件差，有手术禁忌症。

### 7.5 镇痛治疗用药推荐

癌痛治疗原则

1综合治疗；

2从无创性和低危险性方法开始，然后再考虑有创性和高危险性方法。

癌痛综合治疗

1.癌痛综合评估

2.姑息性抗癌治疗及全身性非阿片类/阿片类镇痛药物

3.全身阿片类药物治疗弊大于利时，考虑非侵袭性干预措施

●   恰当姑息性抗癌治疗；

●  加用非阿片类药物；

●  加用辅助药物；

●  应用认知和行为干预措施；

●  借助矫形疗法、其他物理疗法和社会心理干预。

4.全身阿片类药物治疗弊大于利时，考虑侵袭性干预措施

●  区域性止痛技术；

●  神经阻滞术；

●  神经切断术；

●  若上述方法无效时，应用镇静剂等辅助药物协助处理顽固性疼痛。

### 7.6 双膦酸盐治疗推荐

双膦酸盐药物治疗骨转移的用法用量

1.氯膦酸盐片剂1600mg/天，口服给药；或氯膦酸盐针剂300mg/天，静脉注射，> 2小时持续5天，之后换成口服给药；

2.帕米膦酸盐90mg，静脉注射> 2小时，每3-4周重复一次；

3.唑来膦酸盐4mg，静脉注射> 15分钟，每3-4周重复一次；

4.伊班膦酸盐6mg，静脉注射>15分钟，每3-4周重复一次；

伊班膦酸负荷疗法6mg，静脉注射>15分钟，连续三天，持续每3-4周重复一次。

双膦酸盐适应症

1适应症：①骨转移引起的高钙血症；②骨转移引起的骨痛；③ECT异常，X线或CT、MRI证实骨转移；④ECT异常，X线正常，但CT或MRI显示骨破坏；⑤无骨痛症状，但影像学诊断为骨破坏。

2下列情况不推荐使用双膦酸盐：①ECT异常，X先正常，CT或MRI也未显示骨破坏；②存在骨转移风险（LDH或ALP增高）的患者。

双膦酸盐停药指征

1用药过程中检测到与双膦酸盐治疗相关的严重不良反应；

2治疗过程中出现肿瘤恶化，或出现其他脏器转移并危及患者生命；

3继续用药不能获益。

### 7.7 心理干预推荐^[60]^

**Table:** 

轻度焦虑抑郁	临床医护人员给予一些支持性干预 ● 说明诊断、治疗选择和副作用 ● 确保患者理解疾病和治疗选择 ● 推荐适合的患者读物 ● 告诉患者过渡期会更容易感受到痛苦 ● 承认有痛苦 ● 建立信任 进行心理社会评估，明确与癌症照料相关的心理、社会、支持性交流，心理教育，应激管理等；
中度焦虑抑郁	除了上述干预之外，提供心理和/或精神药物治疗； 心理干预依据患者的特点、病程阶段以及癌症相关问题开展；心理教育或认知-行为治疗的问题解决方法适用于疾病的早期阶段，支持-表达性治疗有助于晚期癌症患者
重度焦虑抑郁	● 药物治疗*联合心理社会干预；需提供专业的心理社会服务 ● 镇痛药 ● 抗焦虑药 ● 安眠药 ● 抗抑郁药 ● 支持小组和/或个体咨询 ● 家庭支持和咨询 ● 放松，冥想，创造性治疗
*抗抑郁药应根据患者的症状及药理因素（包括不良反应、耐受性以及潜在的药物交互作用）进行选择。在抗抑郁治疗的第一周内应评估药物不良反应，若出现不良反应，应停药或换药^[61]^。	

附件：

1.主诉疼痛分级（VRS）

**附件1:** 主诉疼痛分级(VRS)

让病人根据自身感受说出，即语言描述评分法，这种方法病人容易理解，但不够精确。具体方法是将疼痛划分为4级：1）无痛2）轻微疼痛3）中度疼痛4)剧烈疼痛，
0级：无疼痛。
Ⅰ级(轻度)：有疼痛但可忍受，生活正常，睡眠无干扰。
Ⅱ级(中度)：疼痛明显，不能忍受，要求服用镇痛药物，睡眠受干扰。
Ⅲ级(重度)：疼痛剧烈，不能忍受，需用镇痛药物，睡眠受严重干扰可伴自主神经紊乱或被动体位。

2.患者一般情况评估（Karnofsky评分）

**附件2:** Karnofsky评分（KPS，百分法）

体力状况	评分
正常，无症状和体征	100分
能进行正常活动，有轻微症状和体征	90分
勉强进行正常活动，有一些症状或体征	80分
生活能自理，但不能维持正常生活和工作	70分
生活能大部分自理，但偶尔需要别人帮助	60分
常需要人照料	50分
生活不能自理，需要特别照顾和帮助	40分
生活严重不能自理	30分
病重，需要住院和积极的支持治疗	20分
重危，临近死亡	10分
死亡	0分
得分越高，健康状况越好，越能忍受治疗给身体带来的副作用，因而也就有可能接受彻底的治疗。等分越低，健康状况越差，若低于60分，许多有效的抗肿瘤治疗就无法实施。	

3. ZPS评分

**附件3:** ZPS评分

0	正常评分
1	症状轻，生活自理，能从事轻体力活动
2	能耐受肿瘤的症状，生活自理，但白天卧床时间不超过50%
3	肿瘤症状严重，白天卧床时间超过50%，但还能起床站立，部分生活自理
4	病重卧床不起
5	死亡

4. WHO癌痛三阶梯止痛

**附件4:** WHO癌痛三阶梯止痛

轻度疼痛：非甾体类抗炎药（NSAID）±辅助药物
中度疼痛：阿片类止痛药+非甾体类抗炎镇痛药±辅助药物
严重疼痛：强阿片类止痛药+非甾体类抗炎药（NSAID）±辅助药物。

5.抑郁、焦虑症状及治疗

**附件5:** 抑郁、焦虑症状及治疗

抑郁	抗抑郁治疗
● 病人感到体力下降，无法坚持下去	
● 病人发出更多的叹息声	● 轻度抑郁可以采用认知和支持性心理治疗或抗抑郁药物治疗
● 抑郁症通常更多见于女性	● 中度至重度抑郁需要抗抑郁药物治疗
● 体力状态不佳的男性肺癌患抑郁发病率高	
● 在戒断尼古丁过程中，容易出现抑郁及过敏易怒	● 社会支持能缓和身体功能的丧失给病人造成的不利影响
● 抑郁会加剧疼痛，如果有抑郁症状，疼痛会成倍加重。	
● 疲劳是抑郁的一个典型表现	● 肺部功能的恢复以及物理治疗是提高机体活力最重要的两种方法
● 睡眠紊乱很常见	
焦虑	抗焦虑治疗
● 对癌症复发转移的恐惧和肺部本身的症状有重叠之处	● 轻度焦虑可采用行为干预，如神经肌肉放松训练
● 主要症状：心悸或者心动过速，呼吸急促，胸痛，恶心和腹部不适，出汗，发抖和震颤，感觉气哽，头晕，感觉异常，寒战或热潮红，现实感丧失，害怕自己失控，还有对死亡的恐惧	● 中度至重度焦虑需要行为干预和抗焦虑药物干预联合应用

6.癌症患者抗焦虑药物

**附件6:** 癌症患者抗焦虑药物

药物	起始剂量	维持剂量
选择性5-羟色胺再摄取抑制剂		
艾司西酞普兰	10-20mg/d	10-20mg/d PO
帕罗西汀	20mg/d	20-60mg/d PO
舍曲林	25-50mg/d	50-150mg/d PO
苯二氮卓类		
阿普唑仑	0.4-0.8mg	PO q6-24h
氯硝西泮	0.5-2.0mg	PO/IM/IVPB q6-24h
劳拉西泮	0.5-2.0mg	PO q4-12h
奥沙西泮	7.5-15mg	PO q6-24h
地西泮	2.5-10mg	PO/IM/IVPB q6-24h
其他药物		
氟哌噻吨美利曲辛	半片-1片	1片/d
曲唑酮	半片	1-2片/d
IM＝肌肉注射；IVPB＝静脉滴注；PO＝口服q＝每		

7.癌症患者抗抑郁药物

**附件7:** 癌症患者抗抑郁药物

药物	起始剂量	维持剂量	注意事项
选择性5-羟色胺再摄取抑制剂			
西酞普兰	10mg/d	20-40mg/d	
艾司西酞普兰	5-10mg/d	10-20mg/d	可能导致恶心、性功能障碍
氟西汀	10-20mg/d	20-60mg/d	半衰期长；可能导致恶心、性功能障碍
帕罗西汀	10mg/d	20-60mg/d	可能导致恶心、镇静
舍曲林	25mg/d	50-150mg/d	可能导致恶心
三环类抗抑郁剂			
阿米替林	25-50mg QN	50-200mg/d	镇静作用较大；抗胆碱能作用；对神经病理性疼痛有效
其他药物			
度洛西汀	20mg/d	60mg/d	可能导致恶心、口干；对神经病理性疼痛可能有效
米氮平	15mg QN	15-45mg QN	镇静，食欲的变化，止吐作用
文拉法辛	18.75-37.5mg/d	75-225mg/d	可能导致恶心；对神经病理性疼痛、潮热症状可能有效
曲唑酮	25mg/d	50-100mg/d	镇静、抗焦虑，抗抑郁作用弱
氟哌噻吨美利曲辛	半片-1片	1片/d	起效快，抗焦虑，抗抑郁作用弱
*抗焦虑、抑郁药应根据患者的症状及药理因素（包括不良反应、耐受性以及潜在的药物交互作用）进行选择。在治疗的第一周内需评估药物不良反应，若出现不良反应，应停药或换药^[64]^。			
